# An Overview of How Epigenetics, MicroRNA-21, and Endocrine Disrupting Compounds Affect Oocyte Maturation and Pre-Implantation Embryo Development

**DOI:** 10.3390/jdb14020028

**Published:** 2026-06-05

**Authors:** Monique Nasser, Reem Sabry, Laura A. Favetta

**Affiliations:** Reproductive Health and Biotechnology Laboratory, Department of Biomedical Sciences, Ontario Veterinary College, University of Guelph, Guelph, ON N1G 2W1, Canada; mnasse02@uoguelph.ca (M.N.); sabryr@uoguelph.ca (R.S.)

**Keywords:** miRNA-21, epigenetics, oocyte maturation, embryo development, endocrine disruptors, fertility

## Abstract

Epigenetic regulation is pivotal in reproductive processes, such as oocyte maturation and pre-implantation embryonic development, and it impacts gene expression without altering DNA sequence through mechanisms including DNA methylation, histone modifications, and non-coding RNAs. Primarily, microRNA-21 is involved in meiotic progression, apoptosis, and cumulus cell function, which are necessary for oocyte competency. miR-21 dysregulation can lead to improper oocyte maturation and poor embryonic development, ultimately causing developmental defects. During pre-implantation embryonic development, DNA methylation and histone modifications contribute to cellular reprogramming, ensuring proper gene activation and repression. Environmentally, endocrine disruptors affect miR-21 expression, potentially disrupting pathways involved in reproductive health and developmental programming. Overall, this review explores the correlation between epigenetics, miRNA regulation, and environmental factors, emphasizing the intricacies of oocyte maturation and pre-implantation embryonic development. This highlights the need for additional mechanistic and translational research in reproductive epigenetics.

## 1. Introduction

Epigenetics focuses on the heritable phenotypic changes without changing the overall DNA sequence [1,2]. These modifications include regulating gene activity and reversibly modifying histones or DNA [3]. This comprises DNA methylation, histone modifications, and non-coding RNA (ncRNA) activity, altering chromatin structure or affecting the transcriptional and translational machinery [4,5]. Notably, epigenetic changes are reversible and can be passed down to offspring, suggesting that environmental factors can have lasting effects on gene expression [6,7,8,9,10]. The dynamic nature of epigenetics allows for further research beyond the traditional genetic framework, opening the door to a broader understanding of how development and disease can be influenced by factors other than the genome itself [6,7,11].

Studies have shown that epigenetic regulation is crucial for genomic stability, cellular differentiation, and gene expression [4]. Additionally, epigenetic mechanisms are responsible for physiological processes, such as immune responses, the onset of cancer, neurological disorders, reproductive function, and aging [12,13,14,15]. Continuous research in the epigenetic field has highlighted how environmental elements, such as diet, pollutants (e.g., Endocrine Disrupting Compounds—EDCs), and stress, can alter the epigenome [8]. These factors can lead to lasting changes in gene expression, influencing health outcomes and disease susceptibility [6,7,11,12,13]. This review covers our current understanding of the role of epigenetic mechanisms in reproductive functions and disease formation, as well as the relationship between gene expression and epigenetics. With this, epigenetic studies provide insights into treatment strategies, effective diagnosis and prognosis, and disease prevention.

### 1.1. DNA Methylation

DNA methylation is a key epigenetic modification that plays a role in gene expression regulation and genomic stability [16,17,18]. It involves the addition of a methyl group to the fifth position of a cytosine residue in a CpG dinucleotide, forming 5-methylcytosine (5mC) [4] by DNA methyltransferases (DNMTs), and is necessary for maintaining development and cell differentiation [16].

Methylation in the gene promoter region is associated with transcriptional repression, whereas methylation within the open reading frame is associated with transcriptional activation [17,19]. Thus, DNA methylation can both repress or activate gene expression, but is most commonly associated with gene silencing. DNA methylation plays a role in biological processes such as genomic imprinting and X-inactivation, the process of silencing one of the two X chromosomes in females, balancing gene dosage [20,21]. Genomic imprinting is the dependence of allelic behaviour on the sex of the parent from which the allele was inherited [21].

### 1.2. MicroRNA

MicroRNAs (miRNAs) are small non-coding RNAs, around 22 nucleotides long, that regulate gene expression by binding to the 3′-untranslated region (3′-UTR) of target messenger RNAs (mRNAs) [22,23,24,25,26], leading to mRNA degradation or translational repression, necessary for modulating biological processes, including development, proliferation, and cellular differentiation [26,27,28,29].

miRNA biogenesis begins in the nucleus, where primary miRNAs (pri-miRNAs) are transcribed by RNA polymerase II just as messenger RNAs (mRNAs) and processed into precursor miRNAs (pre-miRNAs) by a Drosha microprocessor complex [30]. pre-miRNAs are then exported to the cytoplasm by an Exportin-5 protein to be further cleaved by Dicer into miRNA duplexes. A double-stranded RNA attaches to Argonaute, where the two RNA strands dissociate and form single-stranded miRNA-containing RNA-Induced Silencing Complexes (RISCs), necessary for silencing mRNAs [30].

miRNAs exhibit specificity in their interactions with target mRNAs, creating a complex network of regulatory interactions [31] that can destabilize mRNA or inhibit protein translation [32]. miRNAs control stem cell differentiation, development, and disease [32,33,34] and play a role in cell fate specification and differentiation by impacting the expression of transcriptional and molecular factors necessary for embryonic development [35]. For example, miRNAs play a crucial role in the maternal-to-embryonic genome transition (MET), where most maternal transcripts need to be removed for the embryonic genome to take over. miRNAs target maternal transcripts and prevent their translation during MET [36].

In reproduction, miRNAs regulate processes such as oocyte maturation, fertilization, and early embryonic development [37,38,39]. Several miRNAs are involved in proper oocyte development, as shown in Table 1. In oocytes, miRNAs are involved in regulating gene expression patterns for meiotic progression [39,40]. Therefore, their dysregulation can impair oocyte quality and embryonic development, leading to infertility [39,41,42]. miRNAs act as a source of communication between oocytes and cumulus cells, which is necessary for proper oocyte maturation and fertilization [43]. In some cases, miRNAs can also travel from cumulus cells into oocytes through gap junctions, depending on the needs of the oocyte, as transcription in these cells is relatively low. Blocking these connexin-based gap junctions altered miRNA profiles [43]. Overall, these regulatory functions emphasize the importance of miRNAs in maintaining reproductive health [37,38,39].

### 1.3. MicroRNA-21

MicroRNA-21 (miR-21) is a microRNA involved in crucial epigenetic mechanisms, including DNA methylation, apoptosis, oocyte maturation, and cell proliferation [45,47,48,50,71]. miR-21 is particularly important for its impact on various physiological processes such as development, immune response and inflammation, as well as its involvement in cancer and cardiovascular disease [72].

One of the many roles of miR-21 is its regulation of apoptosis [73]. By targeting genes involved in apoptotic pathways, such as the tumour suppressor gene programmed cell death (PDCD4), miR-21 inhibits apoptosis, promoting cell survival [74]. This is crucial for cell development and differentiation, promoting proper tissue growth [75]. Nevertheless, aberrantly increased expression of miR-21 can lead to abnormal cellular survival and disease by preventing the death of damaged cells [76,77,78]. miR-21’s anti-apoptotic function is linked to tumorigenesis, where it supports cancer cell proliferation, invasion, and metastasis [79].

Moreover, miR-21 is upregulated in oocyte maturation, which is necessary for oocyte competence [12,71,80,81]. For example, miR-21 promotes the growth and differentiation of cumulus cells for proper oocyte development [80]. Communication between oocytes and cumulus cells is necessary for oocyte maturation, as cumulus cells secrete factors that impact meiotic maturation and fertilization [82]. Experiments were conducted using the gap junction blocker carbenoxolone to study the miRNA content differences in oocytes and cumulus cells and whether miRNAs were passing through gap junctions. miR-21 was the only known microRNA shown to be differentially expressed within the oocyte with or without functioning gap junctions, proving that it uses this means to enter the oocyte [43]. Ultimately, miR-21 regulates gene expression involved in various cellular communications to improve oocyte competence necessary for fertilization [12,71,80,81,83].

Interestingly, miR-21 upregulation is necessary for oocyte maturation; however, overexpression of miR-21 can induce detrimental effects [77,78]. High levels of miR-21 promote excessive apoptosis of follicular cells, which can impair oocyte development [12,78]. Maintaining miR-21 expression is essential for ensuring oocyte quality and developmental competence, as dysregulation can lead to infertility or developmental defects in the embryo [12]. In cancer cells, miR-21 is upregulated and targets tumour suppressor genes, like PDCD4, diminishing its expression and facilitating cancer cell survival, proliferation, and resistance to chemotherapy [74,84,85]. As a result of its involvement in pathways related to cancer progression, miR-21 can act as a therapeutic factor in modulating its activity [86,87,88,89].

It is important to note that other miRNAs, including Let-7 members, miR-34, miR-155, and many others, may also act in parallel or in opposition with miR-21 by regulating shared pathways including apoptosis, oxidative stress, steroidogenesis, and several signalling pathways [90,91]. Additionally, studies in human follicular fluid report that exposure to oocytes alters these microRNAs as well [92]. Potential antagonistic relationships may also exist; miR-21 is known to be an antiapoptotic regulator, whereas the miR-34 family is associated with proapoptotic regulation [53]. This suggests that a balance between the two is crucial to regulate apoptosis and cell survival. Recent evidence has suggested the phenomenon of miR-miR interactions, which involve the regulation of miRNAs by other miRNAs [93]. This opens up an entirely new avenue of epigenetic research, as the concept of auto-regulation is not yet explored with miRNAs.

### 1.4. Oocyte Maturation

Oocyte maturation is a complex, highly regulated process that prepares the oocyte for fertilization. It involves the resumption of meiosis from prophase I arrest until metaphase II, making the oocyte capable of fertilization [94]. Successful oocyte maturation is essential for oocyte competence and early embryonic development after fertilization [95].

Oocyte maturation is regulated by hormonal signals from the hypothalamic–pituitary–gonadal (HPG) axis [96]. This axis coordinates reproductive function through the secretions of hypothalamic gonadotropin-releasing hormones and subsequent luteinizing hormone (LH) and follicle-stimulating hormone (FSH) secretions, as well as gonadal steroid hormone production [97]. Specifically, LH and FSH are crucial for oocyte meiotic maturation, where LH surge releases oocytes from prophase I arrest to stimulate the resumption of meiosis, and an increase in FSH induces oocyte maturation until metaphase II [94,98,99].

Molecular factors, such as cAMP and miRNAs, are also necessary for controlling oocyte maturation [37,38,39]. Low levels of cAMP are associated with the resumption of meiosis, whereas high levels of cAMP induce germinal vesicle arrest by inhibiting the maturation-promoting factor (MPF) [100,101,102]. miRNAs, including miR-21, are also responsible for oocyte maturation by regulating cumulus cell communication, apoptosis, cell proliferation, and gene expression [12,45,47,48,50,71,82].

Thus, oocyte maturation dysregulation can induce oocyte incompetence or embryonic defects [103]. Moreover, environmental stressors have been shown to affect oocyte quality and meiotic progression. Exposure to pollutants, such as Bisphenol A (BPA), has also been shown to negatively impact oocyte maturation and fertility [104]. Advanced maternal age is also associated with infertility and abnormal oocyte development [105].

### 1.5. Pre-Implantation Embryonic Development

Pre-implantation embryonic development involves a series of stages an embryo undergoes after fertilization before implantation in the uterine wall [106]. This process is essential for establishing an adequate pregnancy. Pre-implantation development includes compaction for cell differentiation and blastocyst hatching for implantation [106]. Moreover, disruptions to these tightly regulated processes can lead to impaired embryonic development, implantation failure, or early pregnancy loss [107,108,109].

After fertilization, the embryo undergoes several divisions, starting with cleavage, without increasing size [106]. These divisions result in the formation of a morula [105]. Then, the transition from the morula to the blastocyst stage is necessary to form specialized cell types and develop embryonic tissues [106]. Additionally, cellular differentiation is characterized by the formation of the inner cell mass (ICM) and the trophoblast layer, which give rise to the fetus and form the placenta, respectively [106].

Four days after fertilization, when the embryo enters the morula stage, compaction begins, where the embryo goes from a loose cluster of cells into a tightly packed mass [106,110,111]. The blastocyst forms a fluid-filled cavity called a blastocoel cavity, expanding as the blastocyst grows [106]. Furthermore, the outer trophoblast layer contributes to the formation of the placenta, and the ICM forms the embryo proper [112,113]. For the embryo to undergo implantation, it must contact the uterine lining, and the blastocyst must hatch from the zona pellucida [106,114].

Since implantation is the interaction between the blastocyst and the endometrium, any disruption to this interaction can lead to pre-implantation failure, leading to pregnancy loss [115]. Thus, processes during pre-implantation development, such as compaction, cell differentiation, and blastocyst hatching, are crucial for successful embryonic implantation in the uterine wall [106,114].

## 2. Discussion

### 2.1. Epigenetic Dysregulation

Environmental factors such as diet, hormones, stress, and EDCs can influence DNA methylation activity [8]. Nutrient intake, such as folate, has been shown to regulate DNA methylation processes [116]. Folate intake influences DNA synthesis, repair, and methylation, with studies showing increased DNA methylation in response to higher folate intake [116,117]. Additionally, folate status serves as an early biomarker for the risk of chronic illnesses, such as cancer, and developmental disorders [116]. Furthermore, exposure to EDCs, such as bisphenol A (BPA), can influence DNA methylation patterns [10]. BPA can induce hypomethylation via estrogen-dependent signalling pathways [10,118,119]. In mice, BPA induced hypomethylation of imprinted genes during oocyte growth while increasing estrogen receptor expression. BPA-induced imprinted gene hypomethylation was eliminated by an estrogen receptor inhibitor, suggesting that the estrogen receptor was the mediator in the process of BPA-induced hypomethylation [120]. Kundakovic and colleagues showed that BPA not only alters estrogen receptor expression, but also mRNA levels of the epigenetic regulators DNMT1 and DNMT3A in the mouse brain, paralleling changes in estrogen-related receptors [121]. In addition, a significant down-regulation in the transcript expression of Igf2 and H19 genes in BPA-resorbed embryos was observed, together with significant hypomethylation of H19 in spermatozoa as well as in resorbed embryos sired by rats exposed neonatally to BPA. These results suggest that disrupted methylation in spermatozoa is passed onto the embryo, causing gene dysregulation, ultimately leading to post-implantation loss, highlighting a possible mechanism of BPA-induced adverse epigenetic effects on fertility [118]. These examples support the long-term effects of environmental exposure on human health, as they can be passed on to future generations [10]. DNA methylation is a dynamic process that can depend on environmental cues and lifestyle choices [8,10]. Importantly, DNA methylation is reversible [122]. DNA demethylases antagonize the reactions catalyzed by DNA methyltransferases [123].

In the context of reproduction and early embryonic development, DNA methylation is also crucial for the regulation of oocyte maturation, embryonic development, and cellular differentiation [97,98,99,100,101,102,103,104,105,106,107,108,109,110,111,112,113,114,115,116,117,118,119,120,121,122,123,124,125]. For instance, DNMT3a is an enzyme required for de novo methylation in gametes [97,126]. It is maternally derived and plays a role in oocyte competency, early pre-implantation development, and cell specification [97,126,127]. DNMT3a is necessary for proper genomic imprinting and gene expression regulation during development [97,126]. Disruptions to these regulatory processes can lead to infertility and developmental defects, demonstrating the importance of DNA methylation regulation in reproduction [128].

miRNA dysregulation has also been associated with cancer, cardiovascular diseases, and neurological disorders [22,129,130,131,132]. Specific miRNAs may act as tumour suppressors depending on their target genes [22]. miR-21 is an example of an oncomiR that promotes cancer progression by targeting tumour suppressor genes and deactivating them [133]. Thus, their impact on gene regulation provides insights into potential biomarkers for disease diagnosis and prognosis [22,87,89,134]. miRNA dysregulation in the brain can lead to neurodegenerative diseases, such as epilepsy and Alzheimer’s, highlighting its role in neuronal homeostasis [135,136].

miRNAs are also involved in immune system regulation [137]. They can modulate immune cell responses, such as infection or injury, by regulating cytokine production [137]. For instance, miRNAs can regulate the activation of T cells, the polarization of macrophages, and dendritic cell activation, important for pro- and anti-inflammatory effects [138,139]. miRNA dysregulation in immune responses can also contribute to autoimmune diseases and chronic inflammation [140,141,142].

The capability of miRNAs has led to their exploration as potential biomarkers for diagnosis and prognosis, as well as their potential use as therapeutic tools [22,87,89,134]. Elevated or reduced levels of specific miRNAs identified in plasma, serum, and urine samples can be indicators of disease and cancer, providing a non-invasive tool for early diagnosis [143,144,145]. This therapeutic potential extends to their roles in reproductive processes, particularly throughout oocyte maturation and early embryonic development.

### 2.2. Epigenetics During Oocyte Maturation

Epigenetic modifications control processes such as gene regulation, chromosome segregation, and developmental competence, which are necessary for fertilization and normal embryonic development [4,146]. Epigenetics regulates gene expression without altering the underlying DNA sequence [4,5]. All epigenetic mechanisms previously mentioned play a crucial role in oocyte maturation [4,5]. Disruptions to these epigenetic processes can lead to developmental disorders or infertility, affecting chromosomal segregation and cell reprogramming, highlighting the importance of epigenetic regulation in reproductive health [12,13,147].

DNA methylation is critical during oocyte maturation due to its role in cell cycle progression, meiotic arrest, and cellular reprogramming for early embryonic development [127,148,149,150]. It is also involved in genomic imprinting, where its dysregulation can cause abnormal embryonic phenotypes and pose a risk of methylation deficiencies [127].

During oocyte maturation, DNA methylation prepares the oocyte for fertilization and embryonic development [124,151]. This modification ensures the silencing of genes during meiotic arrest and activates genes once they are needed for meiotic progression [146,148,149,150]. For example, the methylation of genes, such as cyclins and cyclin-dependent kinases (CDKs), regulates the transition from prophase I arrest to metaphase II, a critical point in oocyte maturation [152]. DNA methylation also plays a role in gene imprinting, expressed in a parent-of-origin fashion where only one copy of a gene is expressed while the other copy is suppressed [153]. Thus, disruptions to DNA methylation patterns of imprinted genes can result in cancer, impaired fertilization, and neurological disorders [128].

Histone modifications play an equally important role in oocyte maturation regulation. Its modifications, including acetylation and methylation, alter chromatin structure, influencing gene expression during oocyte maturation [154]. Histone acetylation, which involves the addition of acetyl groups to lysine residues on histone tails, is correlated with the activation of gene transcription by loosening the chromatin structure into euchromatin [155]. This process is regulated by histone acetyltransferases (HATs) and histone deacetylases (HDACs) [156,157].

In oocyte maturation, histone acetylation is essential for the initiation of meiosis [158]. For instance, acetylating histone H3 at lysine 9 and histone H4 at lysine 16 activates genes required for meiotic progression [159,160]. Atypical histone acetylation regulation, potentially by upregulation or downregulation of HATs or HDACs, can lead to defects in meiotic progression and oocyte competence [156,157]. Moreover, histone methylation is another crucial modification regulating gene expression during oocyte maturation. Methylation of histones can either activate or repress transcription, depending on the lysine residue [161]. In essence, the trimethylation of histone H3 at lysine 4 is associated with activating gene transcription, whereas the trimethylation of histone H3 at lysine 27 is correlated with gene silencing by tightening the chromatin into heterochromatin [162].

Non-coding RNAs, including miRNAs, long non-coding RNAs (lncRNAs), and small interfering RNAs (siRNAs), are necessary regulators of gene expression during oocyte maturation [163]. These molecules are involved in the stability and translation of mRNAs, influencing developmental processes [163].

In addition to miRNAs, lncRNAs also play a key role in oocyte maturation. lncRNAs are longer RNA molecules involved in regulating transcriptional machinery for gene expression [163]. A prime example is lncRNA XIST, which is involved in X-inactivation and has been shown to silence gene expression during oocyte maturation [163]. siRNAs also contribute to gene silencing during oocyte maturation by targeting specific mRNAs for cleavage, translational repression, or destabilization, fundamentally maintaining chromatin structure [163]. These ncRNAs assist with epigenetic stability and prevent transposable element activation, which can lead to genomic instability [163].

Disruptions to epigenetic regulation during oocyte maturation can have drastic effects on reproductive health, leading to developmental disorders, infertility, or miscarriage [164,165,166]. Any irregularity in the epigenetic mechanisms can lead to issues such as improper chromosomal segregation, defects in meiosis, and failed cellular reprogramming during early embryonic development [12,13]. For example, epigenetic dysregulation has led to developmental disorders, such as Prader–Willi syndrome and Angelman syndrome, which are characterized by defects in early embryonic development, leading to infertility in affected individuals [167].

#### miRNA-21 During Oocyte Maturation

miR-21’s role during oocyte maturation is its regulation of meiotic progression. Oocytes undergo meiotic arrest at prophase I and resume meiosis in response to hormonal signals, specifically an LH surge and an increase in FSH, for optimal oocyte competence [94,98,99]. miR-21 regulates meiotic progression by regulating cell proliferation and steroidogenesis, which are crucial for meiotic resumption and oocyte maturation [12,49]. miR-21 knockdown studies in pigs and mice report disrupted meiotic maturation and increased apoptosis of cumulus cells and embryonic arrest at the 4–8 cell stage due to the miR-21 inhibition [47]. In particular, miR-21 targets genes such as phosphatase tensin homolog (PTEN) and PDCD4 [49]. Bioinformatic analysis identified miR-21 as an integral microRNA involved in the miRNA-mRNA co-regulatory networks that may play a key role in controlling post-transcriptomic regulation of the whole ovulatory process [89].

Additionally, miR-21 influences gene expression in cumulus cells for successful fertilization and oocyte competence [12]. Cumulus cells surround the oocyte and play a role in the exchange of molecular signals between the oocyte and the environment [168]. These cells are responsible for oocyte growth, development, and maturation [168]. miR-21 is also highly expressed in cumulus cells and helps to regulate cell proliferation, metabolic activity, and the response to hormonal signals [48,81]. Recent studies in humans aimed to assess the relationship between miR-21 in cumulus cells and human oocyte developmental potential. Higher miR-21 levels were detected in cumulus cells of oocytes that developed into blastocysts compared to cumulus cells of oocytes that did not. In addition, miR-21 was ≈50% less in cumulus cells surrounding oocytes that did not mature or matured to metaphase II but did not fertilize than in oocytes that arrested before reaching the blastocyst stage. Further in vitro studies showed increased apoptosis in cumulus cells with miR-21 knocked down, with a parallel increase in the expression of PTEN, which inhibits the AKT-dependent survival pathway. These data highlight how levels of miR-21 in cumulus cells influence the developmental potential of human oocytes [80].

miR-21 maintains cellular stability during maturation, ensuring the optimal condition of the oocyte for fertilization and embryonic development [169,170]. During oocyte maturation, the cell undergoes various changes, including alterations to the cytoskeleton, membrane potential, and gene expression [171,172,173]. Disruptions to this balance can impair oocyte quality, leading to improper embryonic development [12]. Therefore, miR-21 is important in regulating oocyte quality and early embryonic development [12].

By regulating the expression of genes involved in cell survival, oxidative stress, and metabolic function, miR-21 assists with oocyte stability during maturation [48,81,169,170,174,175,176]. This ensures embryonic viability for fertilization, preventing premature apoptosis and cellular dysfunction [47,80,174]. Therefore, miR-21’s role in cellular homeostasis is crucial for the proper development of oocytes during the maturation process through its role in meiotic progression, apoptosis, and cumulus cell growth and survival [12,49].

Dysregulation of miR-21 activity can lead to defects in the expression of genes such as PDCD4 and PTEN, causing altered follicular development and hormonal signalling [12,49]. Such alterations can lead to impaired oocyte maturation, poor embryonic development, and infertility [12,39,72,177]. Aberrant expression of miR-21 in cumulus cells can trigger aberrant expression of transcripts that play a role in steroidogenesis, impacting the hormonal regulation within these cells. For example, miR-21 is abnormally expressed in the cumulus cells, serum and follicular fluid of patients with polycystic ovarian syndrome (PCOS) [12]. In vitro studies in human granulosa cells showed high levels of miR-21 in patients with PCOS, suggesting it to be a key player in PCOS pathogenesis [178].

### 2.3. Epigenetics During Pre-Implantation Embryonic Development

During pre-implantation development, epigenetic mechanisms, such as DNA methylation, histone modifications, and ncRNA activity, are responsible for cellular reprogramming [179]. These modifications are essential for the transition from a fertilized zygote to a differentiated embryo, with patterns of epigenetic reprogramming enabling the proper activation and repression of developmental genes [4]. Epigenetic regulation is essential for maintaining genomic integrity, where gene expression is tightly controlled, which otherwise may lead to developmental abnormalities [2].

DNA methylation during pre-implantation embryonic development is described as the demethylation and re-methylation of the genome, where both the maternal and paternal genomes undergo reprogramming [180]. Demethylation regulates cellular reprogramming by demethylating enhancers and regulatory elements [181,182]. This is then followed by re-methylation, whereby the epigenetic marks are restored to establish embryonic patterns [181]. These changes are necessary for cell differentiation and proper activation and repression of genes implicated in embryonic development [4].

Genome-wide methylation erasures occur two times throughout development. During embryonic development, when primordial germ cells migrate to the gonadal ridge right before sex differentiation, they need to lose all the methyl marks that turned off specific genes. This is done through the actions of demethylators such as TET1 and TET2. Once sex is determined, new methyl marks will be laid down depending on whether the fetus is male or female; this is the first deletion that occurs. This is done by de novo methylators DNMT3A and DNMT3B. Next, the establishment of either male or female germline entails DNA methylation of sex-specific genes. The next erasure happens after fertilization in the zygote, but this happens more gradually as the embryo develops. A blastocyst will have most methyl marks removed and then re-established after implantation to give rise to a new individual with unique methyl marks [127].

Histone modifications, such as acetylation and methylation, are responsible for further regulating chromatin structure, ensuring genes are activated or silenced during embryonic development [183]. These modifications influence the compaction and accessibility of chromatin, ultimately impacting gene transcriptional activity [184]. Histone acetylation is associated with gene activation by loosening the chromatin structure for transcriptional machinery [155]. Histone methylation, on the other hand, is capable of both activating and repressing transcription [161]. As mentioned, when histone H3 at lysine 4 is trimethylated, gene transcription is activated [162]; however, when histone H3 at lysine 27 is trimethylated, gene transcription is silenced [162]. The role of histone methylation in reproduction is its ability to maintain a pluripotent state for early embryos [184]. Thus, chromatin remodelling processes are crucial for proper gene expression during embryonic development, as they facilitate cellular differentiation and progression.

Additionally, ncRNAs contribute to proper pre-implantation embryonic development by modulating gene expression and ensuring proper cell differentiation and proliferation [185,186]. ncRNAs, including miRNAs and lncRNAs, play a role in modulating mRNA translation and chromatin structure [163,187,188].

Specifically, lncRNAs are long, non-coding RNA molecules that do not code for proteins but regulate gene expression through chromatin remodelling, transcriptional interference, alternative splicing, cell differentiation, and cell cycle regulation [22,26,28,29,189,190,191]. Together, ncRNAs contribute to the overall gene expression required for pre-implantation embryonic development. They ensure proper cell differentiation and tightly regulated transitions between cell stages [4].

Epigenetic regulation is crucial for proper pre-implantation embryonic development, where disturbances to these processes can lead to developmental defects and disease [192]. Abnormalities in DNA methylation, histone modifications, or ncRNA activity can alter cellular reprogramming and the activation or repression of transcriptional activity [179,184]. Epigenetic errors during embryonic development can lead to impaired implantation, genetic disorders, and developmental disorders [164,165,166]. Therefore, understanding the epigenetic regulation of events during this window of development offers insights into fertility, cell differentiation, and potential therapeutic strategies for early developmental disorders [13].

#### miR-21 During Pre-Implantation Embryonic Development

miR-21 is a necessary regulator of gene expression during pre-implantation embryonic development, influencing processes such as cell proliferation, differentiation, and apoptosis [71,83]. miR-21 exerts its effects by binding to the 3′-UTR of target mRNAs, regulating their stability and the translation of proteins [22,26,193]. miR-21’s tightly controlled regulation maintains the balance between cell survival and programmed cell death, which is essential for normal embryonic development to remove any damaged cells [73].

Additionally, miR-21’s role in gene expression is important for determining cell fate [194]. During pre-implantation, cells must undergo specific reprogramming mechanisms to differentiate into various cell types to form tissues and organs of the embryo. miR-21 regulates several genes involved in signalling pathways, specifically the mitogen-activated protein kinase (MAPK) signalling pathway, as well as genes related to cell cycle regulation and apoptosis [195]. The MAPK pathway mediates responses to external signals and stress, and it plays a role in determining cell fate [194]. It does this through transmitting growth factors and stress signals to regulate cell proliferation, differentiation, survival, and apoptosis [196]. One study showed that miR-21 activates MAPK signaling indirectly through its targeting of DUSP8, a phosphatase that suppresses MAPK pathway activity [197]. When miR-21 was upregulated, DUSP8 decreased and MAPK pathway phosphorylation increased, which promoted endothelial apoptosis [197]. Therefore, miR-21’s modulation of the MAPK pathway helps maintain proper embryonic development by controlling cell differentiation and proliferation.

Furthermore, miR-21 is involved in the regulation of key transcription factors, impacting cellular differentiation and somatic reprogramming of cells to a pluripotent state [83,198]. It is directly involved in cell cycle regulation and apoptosis, especially as cells progress through each phase of division to generate new cells [83]. miR-21 regulates these transitions, ensuring the balance between cellular apoptosis and proliferation during pre-implantation development [73].

In cell differentiation, miR-21 regulates transcription factors to determine cell fate and ensure proper activation of genes. During pre-implantation development, tightly controlled gene expression is necessary for proper cell differentiation and organ formation [72,73]. During somatic cell reprogramming, miR-21 helps maintain embryonic capacity for differentiation [199]. miRNA regulation and pluripotency are crucial for pre-implantation embryonic development, where miR-21 ensures that cells can differentiate into any cell type [199]. Research on miR-21’s ability to enhance somatic reprogramming of cells to a pluripotent state during embryonic development has also been investigated concerning induced pluripotent stem cell (iPSC) generation, a new sector of regenerative medicine [199,200].

Thus, disruption of miR-21 expression during early embryonic stages has been linked to poor embryonic development, highlighting its importance in gene expression [103]. Any alteration to miR-21’s expression can lead to consequences in embryonic viability and successful implantation [201]. Furthermore, miR-21’s mechanisms of action during pre-implantation have provided opportunities for exploring its role in chemotherapeutics [202,203]. For example, miR-21 has been shown to influence drug resistance and cell survival in response to chemotherapies [202,203]. Therefore, miR-21’s roles in embryonic development and cancer provide insights into reproductive health issues [177]. Understanding miR-21 cellular processes during pre-implantation development can help mitigate embryonic defects and supply better diagnostic strategies. Despite the important regulatory function of miR-21, its expression profile and associated downstream signaling pathways remain susceptible to environmental disruptions, particularly through EDCs.

### 2.4. Effect of Endocrine Disruptors on miRNA-21 During Oocyte Maturation and Pre-Implantation Embryonic Development

Endocrine disruptors are environmental chemicals that interfere with hormonal systems, showing an impact on miRNA regulation during reproductive development, including oocyte maturation and pre-implantation embryonic development [104,204,205]. Endocrine disruptors, like BPA, have been extensively studied, and their molecular mechanisms of action have been reported. In the classical pathways, BPA is most commonly known to interact with the estrogen receptor, which can translocate to the nucleus and interfere with gene expression. BPA can also enter the cellular membrane and increase the production of reactive oxygen species (ROS), which can induce DNA damage. A significant decrease in antral follicles in parallel to a rise in atretic and cystic follicles in the ovaries of BPA-treated rats was observed in parallel to a decrease in catalase, superoxide dismutase, and peroxidase levels and an increase in ROS, suggesting that BPA exposure during the pre-pubertal stage has the potential to induce oxidative stress [206]. BPA, and its analogs BPS and BPF, increased the production of ROS and the levels of antioxidants in bovine granulosa cells [206]. BPA only increased ROS production in oocyte complexes and decreased the levels of antioxidant enzymes, supporting BPA’s ability to alter oxidative stress in oocytes [207]. Human studies show that EDCs are detectable in follicular fluid and associated with poor fertility outcomes [92,208]. These outcomes included fewer oocytes retrieved, fewer mature oocytes, and a decrease in high-quality embryos [92,208].

**Figure 1:**
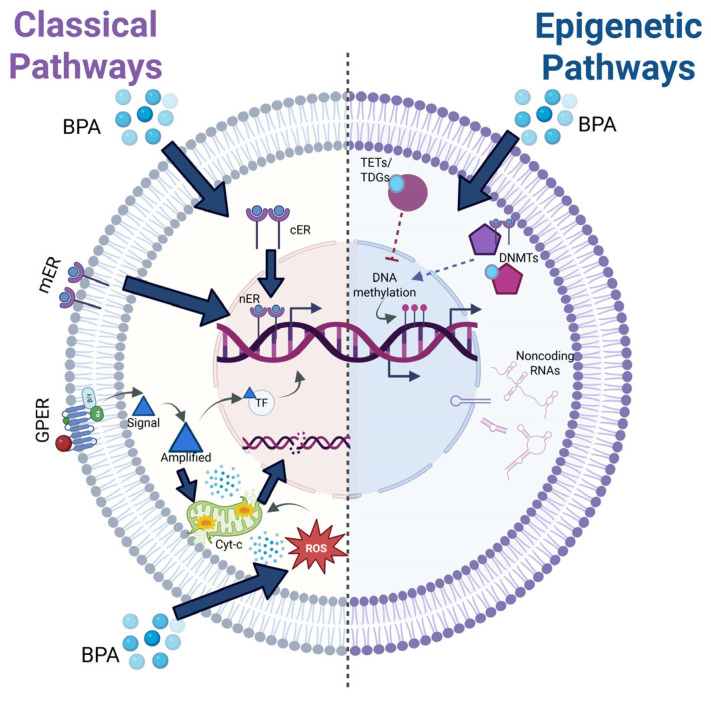
Classical and epigenetic consequences of BPA. Endocrine disruptors such as BPA can induce molecular changes through classical pathways by binding to estrogen receptors, either membrane (mER), cytosolic (cER), or G-protein-coupled estrogen receptors (GPERs). It can also increase reactive oxygen species (ROS) and alter transcription factors (TFs) or induce DNA damage. Alternatively, they can induce changes in epigenetic pathways by altering DNA methylation genes such as DNMTs or TETs/TDGs or by altering the expression of non-coding RNAs. [Created in https://BioRender.com].

Alternatively, newer studies have shown that BPA can alter epigenetic pathways by altering the expression of genes responsible for DNA methylation and histone modifications and also by altering the expression of ncRNAs (Figure 1). In Zebrafish, BPA down-regulated oocyte maturation-promoting signals, likely through changes in the chromatin structure mediated by histone modifications, and promoted apoptosis in mature follicles [209]. BPA, but not BPS, dysregulated the expression of target microRNAs during oocyte maturation in bovine, with a significant increase in miR-21, miR-155, and miR-29a and a decrease in miR-34c and miR-10b, indicating that BPA effects are likely miRNA-specific rather than a global effect on miRNA synthesis and processing mechanisms and that its analog, BPS, may not possess the same properties required to interfere with these miRNAs during bovine oocyte maturation [105]. Other EDCs include reports that show that urinary phenol and phthalate metabolites were also found in human follicular fluid and linked to altered miRNA profiles, suggesting a link between EDCs, epigenetic regulation, and oocyte development [210].

microRNAs, including miR-21, are prone to environmental disturbances that can affect cellular functions [211]. During oocyte maturation, miR-21 is implicated in regulating processes such as apoptosis, cell cycle progression, and embryonic development [44,45,46,47,48,71,83]. miR-21 has an important role in oocyte quality and proper embryonic development. Disruptions to miR-21 expression, particularly by environmental pollutants, can lead to abnormal development and infertility [71].

Several endocrine disruptors, such as bisphenol A (BPA), phthalates, and pesticides (DDT), alter miR-21 expression, causing an imbalance in essential processes, like hormonal signalling pathways [96,103,212,213]. Both BPA and DDT were shown to decrease miR-21 expression in breast carcinoma cell lines, but their effect was inverted if the estrogen receptor was down-regulated, strengthening that EDCs, such as BPA and DDT, may likely act independently of the estrogen receptor [213]. Fenhexamid and fludioxonil, antifungal agents used as pesticides in agricultural applications, increased miR-21 in a dose-dependent manner and reduced the expression of miR-21 target PDCD4 in breast cancer cell lines. Fenhexamid and fludioxonil did not affect androgen-induced miR-21 expression [212]. BPA is an EDC with a major known impact on miR-21 activity as it increases its expression in oocytes and cumulus cells, inducing the downregulation of methylation processes, ultimately resulting in hypomethylation [71,214]. Hypomethylation consists of the failure to methylate groups on DNA and histones, resulting in activated genes that were meant to be silenced, thereby altering gene expression and genomic stability [16,214]. This is in line with the observation of increased miR-21 expression when methylation was inhibited during the morula–blastocyst transition in mice [33].

This review focuses on Bisphenol as an EDC model since it is the most common in the literature that investigated how EDCs regulate microRNA function within a reproductive context. However, within bisphenols, a wide class of alternatives has been tested, including BPS and BPF, and it is worth noting some differences. A study found that BPA alters the expression of key miRNAs in a different manner than BPS [103]. Generally, studies that have investigated both BPA and BPS also report that they act through different molecular pathways [215]. This suggests that BPA and its alternatives should not be thought of in the same manner, as they can act through different mechanisms.

Studies linking EDC exposure to miR-21 dysregulation and subsequent epigenetic regulations are relatively few, as this is a new complex avenue for environmental and molecular research. Most studies discussed represent correlative changes seen in models where both systems are evaluated. One study by Sabry et al. [71] proves that there is direct causal evidence between EDC-induced miR-21 changes and DNA methylation in bovine granulosa cells [71]. This study inhibited miR-21 in bovine granulosa cells, treated them with BPA and measured the levels of 5′ methylcytosine (5mC). BPA treatments alone resulted in decreased 5mC levels, supporting studies that have reported hypomethylation after BPA exposure [104,206]. When miR-21 was inhibited, this effect was reversed [71]. The study indicates that miR-21 inhibition resulted in an increase in DNMT1 and DNMT3A, which could explain the previous observation. Other studies are more correlational; for example, exposure to the phthalate DEHP is linked to histone acetylation changes in rat brains [216], and miR-21 is shown to target histone deacetylase 8 in A549 cells [217].

**Figure 2:**
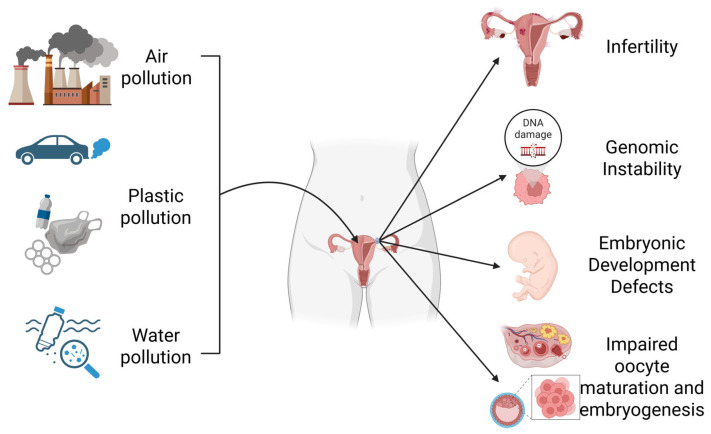
Impact of environmental pollutants on reproductive health in females. Environmental pollutants impact the reproductive health of females, causing infertility, genomic instability, embryonic developmental defects, and impaired oocyte maturation. Pollutants from air, plastics, and water can affect the overall health of the reproductive system. [Created in https://BioRender.com].

The opposite regulation has also been reported; hypomethylation, caused by BPA exposure, has been shown to upregulate miR-21 expression in tumours, as overexpression of miR-21 has been directly correlated with the growth, invasion, and metastasis of tumour cells [218]. Thus, miR-21 dysregulation due to BPA exposure can lead to genomic instability, impaired oocyte maturation, abnormal fertilization, and defective embryonic development at the pre-implantation stage (Figure 2) [71,214]. Furthermore, dysregulation of miR-21 by endocrine disruptors has also been shown to affect key signalling pathways, including the MAPK and PI3K pathways, which are crucial for cellular proliferation, differentiation, and survival [219]. The PI3K pathway mainly supports cell survival, metabolism, and resistance to apoptosis [220]. Recently, Lam et al. reported a novel role of BPA in the regulation of Exportin-5 that led to dysregulation of microRNA biogenesis through miR-21 in ovarian cancer cells, where BPA has been shown not to act through the traditional estrogen receptor pathway [221].

As discussed throughout this review, miR-21 has a crucial role in oocyte maturation and is ultimately susceptible to environmental disturbances. This reflects the influence of endocrine disruptors on miR-21, demonstrating its consequences on reproductive health. Endocrine disruptors have been shown to cause endocrinological dysfunction, developmental abnormalities, and infertility [12]. Prenatal exposure can specifically lead to defects in fetal development, genetic mutations, or congenital disabilities [222]. In mice, prenatal exposure to the anti-androgenic compound vinclozolin caused transgenerational effects on germ cells. A reduction in the number of embryonic primordial germ cells and an increased rate of apoptotic cells, along with a decrease in fertility rate, were observed in F1 to F3 generations. miR-21 expression was deregulated in three successive generations of mice, suggesting that embryonic exposure to environmental endocrine disruptors induces transgenerational epigenetic deregulation of expression of microRNAs affecting key regulatory pathways of germ cell differentiation [223]. Understanding the correlation between endocrine-disrupting compounds and miR-21 during oocyte maturation and early embryonic development is crucial for assessing reproductive health risks associated with environmental effects.

Another important note is that, in addition to environmental exposure, advanced maternal age is also associated with epigenetic dysregulation, impaired oocyte quality and changed in miRNA expression profiles [224]. The interaction correlations between age, miRNA dysregulation and EDC exposure remains to be elucidated, but these factors may contribute together to reproductive decline and warrant further research.

The current literature is limited in that there is direct evidence that EDC exposure can alter miR-21 and that miR-21 can influence other epigenetic mechanisms, such as DNA methylation and histone modifications, but any direct evidence that EDC-induced miR-21 changes specifically drive changes in DNA methylation and histone modifications is in its infancy and presents a new avenue of research into epigenetic cross-talk.

## 3. Therapeutic and Intervention Strategies

Since miR-21 regulates several pathways, including apoptosis, proliferation, and inflammation, it has presented a promising therapeutic target in several disease models. Intervention models include miR-21 mimics, antagomirs, and LNA inhibitors to help regulate miR-21 activity. These are largely restricted to therapies for cancer and fibrosis models [72,76]. Current therapeutic approaches with microRNAs focus mainly on inhibiting them in order to restore signals for tumour suppression [76]. For example, Matarlo et al. [225] found that blocking miR-21 restored its downstream target, PDCD4, decreasing the growth of cancer cells. In Fibrosis, miR-21 is also considered a therapeutic target due to its role in inducing fibroblast activation and profibrotic signalling pathways. One study showed that miR-21 inhibition helped to reduce hypertrophic scar formation in cardiac fibrosis [226].

Although there are few, there are also studies that have looked into miR-21 as a therapeutic intervention for reproductive abnormalities [227,228]. One study showed that miR-21-loaded exosomes have preclinical promise to improve ovarian function in cases of autoimmune premature ovarian insufficiency [227]. Another study showed that these same miR-21 exosomes can help to inhibit apoptosis in granulosa cells, which would also help to improve factors that contribute to premature ovarian insufficiency [228]. Overall, miR-21-based therapies remain at the preclinical stage, and future studies could expand on these approaches.

## 4. Limitations and Future Directions

Current limitations and challenges for this complex, intertwined system are the difficulty in establishing causality between EDC-induced changes in miR-21, followed by downstream disruptions in epigenetic mechanisms and even further into impaired oocyte and embryonic development. The best way future studies can address this is by utilizing current research tools, including CRISPR-based approaches, rescue experiments, mimics, and antagomirs, to uncover whether influencing miR-21 regulation plays a role in normal epigenetic patterns and whether this influence can improve oocyte maturation.

Another potential limitation observed is the limited relevance of current models to human fertility. The use of animal models and in vitro granulosa cell cultures does not fully represent real conditions. For example, animal studies allow controlled EDC dosing and direct oocyte manipulation, whereas human studies are restricted by access to samples. Future research should focus on more relevant models, such as cumulus–oocyte complexes or human follicular fluid, to study this model and allow it to be more translatable to human fertility.

## 5. Conclusions

Epigenetic regulation is crucial for oocyte maturation and pre-implantation embryonic development to ensure proper gene expression, cellular differentiation, and genomic stability. Its mechanisms, including DNA methylation, histone modifications, and non-coding RNA activity, all play critical roles in controlling embryonic progression. Specifically, miR-21 regulates cell differentiation, proliferation, and apoptosis during early embryonic development. Dysregulation of these processes can lead to developmental defects, infertility, and neurological disorders. Moreover, environmental factors, especially endocrine-disrupting compounds, such as BPA, phthalates, and pesticides, can impact miR-21 expression, causing impaired oocyte maturation and embryogenesis.

Currently, the evidence that supports correlations between epigenetic cross-talk involving miR-21 under EDC exposure and several mechanistic questions is still in its infancy. Future research should focus on the precise molecular pathways through which miR-21 interacts with methylation modulators and how that impacts developmental pathways.

Overall, this review highlights the role of epigenetics in early embryonic development and miR-21’s sensitivity to endocrine disruptors. By understanding the relationship between miR-21 and external factors, improvements to fertility and therapeutic approaches to reproductive disorders can be made. This review is beneficial in the field of reproductive medicine and environmental sciences by highlighting strategies to improve research approaches with a potentially larger impact on oocyte quality and viability. Therefore, studying the correlation between epigenetic regulation during oocyte maturation and embryonic development is essential to improving fertility treatments, diagnosing reproductive disorders, and promoting healthy pregnancy outcomes.

## 6. Methodology

This literature review was conducted through searching for articles on PubMed and Google Scholar databases. Articles published between 2010 and 2026 were prioritized. The literature strategy used was to focus on recent studies on epigenetic regulation, miR-21 function, endocrine-disrupting compounds (EDCs), oocyte maturation, and pre-implantation embryonic development. Search terms included combinations of the aforementioned topics, and articles were chosen based on relevance to reproductive epigenetics, miRNA regulation, and EDC-linked reproductive outcomes.

## Figures and Tables

**Table 1:** miRNAs and their importance to oocyte development.

miRNA	Role of Regulation	mRNA Targets	References
miR-21	Oocyte maturation, cell proliferation, and apoptosis	PTEN, PDCD4	[44,45,46,47,48,49,50,51]
let-7b	Steroidogenesis, cell proliferation, and apoptosis	HMGA2, CDC25A	[45]
miR-34	Cell cycle progression, cell proliferation, oocyte maturation	BCL-2, Notch	[52,53]
miR-375	Oocyte maturation, cell proliferation, and apoptosis	YAP, MYC	[54,55,56,57]
miR-151	Oocyte quality, oocyte maturation, and embryonic development	LRCH1, FZD5	[58,59]
miR-133	Oocyte maturation and oocyte growth	GNL2, BCLAF1	[60,61,62]
miR-224	Granulosa cell proliferation and function	SMAD4, Ptx3	[63,64,65]
miR-145	Oocyte competence and granulosa cell proliferation	SOX2, OCT4	[66,67]
miR-429	Fertility and ovulation	CHMP5, CFL2	[68,69,70]

## Data Availability

All figures created in Biorender.com with a valid publication and licensing rights.

## Abbreviations

The following abbreviations are used in this manuscript:

ncRNA	Non-coding RNA
EDC	Endocrine Disrupting Compound
5mC	5-methylcytosine
DNMT	DNA Methyltransferase
BPA	Bisphenol A
miRNA	MicroRNA
mRNA	Messenger RNA
Pri-miRNA	Primary microRNA
Pre-miRNA	Precursor microRNA
3′UTR	3′ Untranslated Region
RISC	RNA-induced Silencing Complex
MET	Maternal-to-Embryonic Genomic Transition
PDCD4	Programmed Cell Death
HPG	Hypothalamic Pituitary Gonadal
LH	Luteinizing Hormone
FSH	Follicle Stimulating Hormone
MPF	Maturation Promoting Factor
ICM	Inner Cell Mass
CDK	Cyclin-Dependent Kinases
HAT	Histone Acetyltransferase
HDAC	Histone Deacetylase
lncRNA	Long noncoding RNA
siRNA	Small interfering RNA
PTEN	Phosphatase Tensin Homolog
PCOS	Polycystic Ovarian Syndrome
MAPK	Mitogen-Activated Protein Kinase
iPSC	Induced Pluripotent Stem Cells
ROS	Reactive Oxygen Species
mER	Membrane Estrogen Receptor
cER	Cytosolic Estrogen Receptor
GPER	G-protein-Coupled Estrogen Receptor
TF	Transcription Factor
